# Assessing the ECHO^®^ model's role in strengthening health department responses to dementia risk

**DOI:** 10.3389/fpubh.2025.1686733

**Published:** 2026-01-05

**Authors:** Shelby Sutton Roberts, Dana Sohmer, Nia White, Jessica M. Potts, Rachel Goldberger, Mickal Lewis, Chelsea Kline, Lillian Madrigal, Rochelle Roberts, Nikki Lawhorn Rider

**Affiliations:** 1 Division of Programs, Alzheimer's Association, Chicago, IL, United States; 2 Building Our Largest Dementia Infrastructure (BOLD) Public Health Center of Excellence on Dementia Risk Reduction, Alzheimer's Association, Chicago, IL, United States; 3 Department of Behavioral, Social, and Health Education Sciences, Emory University Rollins School of Public Health, Atlanta, GA, United States; 4 Tennessee Department of Health, Nashville, TN, United States

**Keywords:** Alzheimer's, Alzheimer's disease and related dementias, dementia risk reduction, ECHO, workforce capacity

## Abstract

**Introduction:**

The Alzheimer's and Dementia Care ECHO Program was adapted for Public Health Professionals and a pilot was conducted among professionals from state and local health departments in Tennessee. The series aimed to increase knowledge, confidence, and public health action around dementia risk reduction across six virtual sessions, which featured a brief presentation followed by a case discussion.

**Methods:**

The evaluation sought to understand the ECHO's impact on public health professional practice and knowledge change regarding dementia risk reduction using a mixed-methods approach. Data were collected through pre and post-series surveys, surveys following each session, and focus groups.

**Results and discussion:**

Participants reported high levels of satisfaction with the ECHO series, increased recognition of dementia risk reduction as a public health issue, and increased knowledge and confidence about reducing dementia risk. Overall, 70% of respondents said they would implement something they learned immediately or in the next 30 days. Increased recognition of and confidence to implement dementia risk reduction strategies can lead to improved health outcomes and more proactive measures being integrated into public health strategies.

## Introduction

The Building our Largest Dementia Infrastructure (BOLD) for Alzheimer's Act, first passed in 2018 and reauthorized in 2024, directs the CDC to establish Alzheimer's Centers of Excellence, increase data analysis and timely reporting, and provide funding for the CDC to distribute to health departments across the country (P.L.118–142). The BOLD Act, and guidance from the Healthy Brain Initiative Road Map, set up the structure and resources for health departments to take a public health approach to Alzheimer's disease and other dementias. Further supporting this public health approach to dementia, the Centers of Excellence established by the BOLD Act are used by health departments as hubs of information, training and support on risk reduction, early detection, and caregiving.

A critical piece to achieving success with a public health approach to Alzheimer's disease and other dementias is ensuring there is a diverse and skilled workforce (1). The BOLD Public Health Center of Excellence on Dementia Risk Reduction (Center) was established in 2020 at the Alzheimer's Association. The Center is tasked with translating evidence on dementia risk into information health departments can use in practice and ensuring health departments are knowledgeable and confident in their ability to implement dementia risk reduction activities. The Center collaborated with the Alzheimer's Association Project ECHO Superhub to develop a training model aimed at enhancing the knowledge, confidence, and collaboration of state and local health departments in dementia risk reduction.

The Alzheimer's Association adopted the Project ECHO^®^ (Extension for Community Healthcare Outcomes) model in 2018 and since then has used telementoring to train and support thousands of healthcare professionals across the care continuum. Alzheimer's Association Project ECHO leverages technology to connect dementia experts with front-line providers, expanding access to early and accurate diagnosis, effective management, and comprehensive support for individuals and families affected by Alzheimer's disease and other dementias. In 2024, the Alzheimer's Association was designated an ECHO Superhub, enabling it to provide training and mentorship to organizations adopting the model, with the goal of expanding the reach of Project ECHO in dementia and improving access to high-quality care for more individuals.

Project ECHO programs have proven effective in helping clinicians with cognitive assessments, the diagnostic process, and management of disease (2–6) as well as with specific aspects of care, including managing behavioral symptoms (7) and pain (8). Beyond clinical training, these programs foster a supportive, interprofessional learning community that encourages shared experiences and case-based learning–especially valuable for healthcare professionals who often work in isolation (4, 6, 9).

Building on this strong foundation, the Alzheimer's Association ECHO Superhub and the Center partnered to develop a specialized ECHO program focused on training public health professionals at local and state health departments to understand dementia risk factors and integrate brain health strategies into existing public health initiatives.

## Materials and methods

### Program purpose

The purpose of the program is to refine the application of the ECHO model for public health professionals and explore its potential for replication in other states. The project had three goals: (1) increase public health professionals' knowledge about dementia as a public health issue; (2) strengthen public health professionals' capacity to implement activities related to dementia risk reduction; and (3) stimulate change in public health practice related to dementia.

### Program description and setting

Launched in 2022, the Alzheimer's and Dementia Care ECHO Program for Public Health Professionals is a collaborative effort between the Alzheimer's Association's Project ECHO and the Center and began with developing a curriculum that aligned with the Healthy Brain Initiative (HBI) Road Map to support state-level dementia risk reduction efforts.

Unlike traditional webinars, the ECHO model uses a dynamic, mentor-guided format that fosters interactive learning. It enables public health professionals to engage directly with experts and apply real-world scenarios to address the challenges and opportunities in dementia risk reduction.

The Association convened subject matter experts (collectively known as the “Hub”) and local and state health department staff to form virtual learning networks and engage in collaborative problem solving. The ECHO program is designed to occur within one state so communities and resources can be leveraged, and the final session can be tailored to that state's priorities. The sessions of the ECHO were designed to educate public health professionals on risk factors and social determinants of health that contribute to dementia risk and provide practical application opportunities to improve brain health in their communities. In addition to knowledge sharing, bringing together state and local health departments allows for resource sharing and networking.

The six-session ECHO series, held from February 13, 2024 to March 19, 2024, took place through weekly, hour-long video conferences. Each session featured a brief presentation by the a Hub member on a key aspect of dementia risk reduction, followed by a case discussion where participants presented specific challenges to risk reduction activities in their health departments. The case discussions were designed to foster dynamic exchanges, with clarifying questions and actionable recommendations from the group. This collaborative, problem-solving format exemplified the ECHO “all teach, all learn” model, where every participant contributes to the learning experience. Session topics and participant attendance details are provided in Table 1.

**Table 1:** The Alzheimer's and Dementia Care ECHO program for public health professionals session topics and participants.

**Date**	**Session topic**	**Number of participants**	**Number of health departments represented**
2/13/24	Introduction to Project ECHO and Dementia Risk Reduction	84	32
2/27/24	Leveraging Data to Promote Risk Reduction	77	33
3/5/24	Engaging Community Partners in Risk Reduction Activities	69	34
3/12/24	Effective Dementia Risk Reduction Communications and Messaging	79	34
3/19/24	Action Planning to Reduce Dementia Risk	76	34
	**Average**	77	33

Emory Centers for Public Health Training and Technical Assistance (Emory Centers) conducted the evaluation of the ECHO series to identify opportunities for program improvement and support the replicability of the program in other states. The mixed-methods evaluation approach included programmatic data analysis and primary data collected from participants and Hub members through surveys and focus groups.

### Measures and variables

Emory Centers administered pre- and post-series surveys using unique Qualtrics links to track individual responses over time.

### Pre and post-series surveys

The pre-series survey collected standard Alzheimer's Association ECHO demographic and organizational data (e.g., birth year, gender, race/ethnicity) while also establishing a baseline for participants' knowledge, beliefs, and confidence related to ADRD as well as risk reduction strategies. The post-series survey measured changes in these areas and captured participants' experiences in the program, including whether they adopted new practices in their state and local health departments due to their ECHO participation.

The pre-series survey was open from February 1 to 16, 2024. The post-series survey was initially open from March 22 to 29, 2024, and reopened from April 3 to 12, 2024 to improve response rates. To assess changes in knowledge, beliefs, and confidence among public health professionals, a paired sample *t*-test was conducted using data from participants who completed both surveys (*n* = 60).

To understand public health professionals' perspectives on the importance of dementia risk reduction, participants were asked to rate their agreement using a 5-point Likert scale (from 1: strongly disagree to 5: strongly agree) with two statements: (1) dementia risk reduction is a public health issue; and (2) dementia risk reduction plays a role in public health.

### Post-session surveys

The evaluation also included post-session surveys for each of the six sessions to assess satisfaction with each session, how likely participants would be to share the information they learned, and any new knowledge that was gained. Post-session Qualtrics surveys were shared via email following each session and remained open for 7 days following the session.

### Focus groups

Emory Centers hosted a virtual post-program focus group with ECHO Series Hub members a week following the conclusion of the program in March 2024. The focus group lasted 49 minutes and included five participants. To reduce the potential for bias, Alzheimer's Association staff did not participate in the focus group. The focus group inquired about (1) challenges and successes, (2) perceived knowledge and readiness levels of session participants, and (3) considerations for improving future ECHO series. Analysis occurred in April using rapid qualitative assessment. Rapid qualitative analysis is a method used to analyze thoughts, opinions, and experiences that participants share in focus groups and is best used when a project needs to draw data quickly from multiple sources, especially when that data needs to be triangulated with quantitative data.

In July 2024, 3 months after the ECHO Series concluded, the evaluation team invited all ECHO participants to a follow-up focus group. The focus group was conducted using a convenience sample of six participants. The 51-min session aimed to gather deeper insights into participants' experiences and explore the potential impacts of the ECHO Series. Discussion topics included: (1) participant successes and challenges; (2) levels of knowledge, reach, and implementation; and (3) suggestions for improving future ECHO sessions. Focus group data were analyzed using a rapid qualitative assessment approach.

## Results

### Pre-series survey

In total, 101 individuals from 38 health agencies registered for the ECHO series. Of those who registered, 74 completed the pre-series survey (73% response rate). Demographics of registrants are included in Table 2. On average, pre-series survey respondents reported seven years of experience in public health and spent an average of seven years in their current position and at their current organization. Additionally, most respondents reported working at their local/regional (*n* = 44; 60%) or state health department (*n* = 29; 39%). Just under a third reported holding a nursing position (*n* = 23; 31%). Over a fifth of respondents reported working as a health educator (*n* = 17; 23%). Nearly all individuals were female (*n* = 65; 88%), white (*n* = 57; 77%), and identified as not being of Hispanic, Latino/a/x, or Spanish origin (*n* = 69; 93%).

**Table 2:** ECHO participant demographics (*n* = 74).

**Characteristic**	***n* (%)**
**Job title**	
Health educator	17 (23.0)
Unknown	11 (14.9)
Registered nurse	9 (12.2)
Nursing supervisor	8 (10.8)
County director	4 (5.4)
Public health office coordinator	4 (5.4)
CHANT social worker	3 (4.1)
Licensed practical nurse	3 (4.1)
Public health office assistant	3 (4.1)
Disease intervention specialist	2 (2.7)
Nutritionist	2 (2.7)
Advanced practice nurse	1 (1.4)
CHANT coordinator	1 (1.4)
Epidemiologist II	1 (1.4)
Licensed professional counselor	1 (1.4)
Nurse practitioner	1 (1.4)
Physician	1 (1.4)
Program director	1 (1.4)
Public health nurse	1 (1.4)
**Organizational affiliation**	
Local/regional health department	44 (59.5)
State health department	29 (39.2)
Public health organization	11 (14.9)
Healthcare organization	2 (2.7)
Other	2 (2.7)
Aging services network or program	1 (1.4)
University or college	1 (1.4)
**Educational attainment**	
Bachelor's degree	37 (50.0)
Some college/associate degree	18 (24.3)
Post/Professional degree	16 (21.6)
High school	1 (1.4)
Missing	2 (2.7)
**Gender**	
Female	65 (87.8)
Male	2 (2.7)
Prefer not to say	3 (4.1)
Missing	4 (5.4)
**Racial identity**	
White	57 (77.0)
Black or African American	12 (16.2)
Prefer not to say	2 (2.7)
American Indian or Alaska Native	0 (0.0)
Asian	0 (0.0)
Native Hawaiian or Other Pacific Islander	0 (0.0)
Another race, ethnicity, or origin	0 (0.0)
Race not listed	0 (0.0)
Missing	3 (4.1)
**Characteristic (*****n** =* **71)**	**Average**	**Standard deviation**
Number of years in current position	6.95	7.40
Number of years with current agency (any position)	6.96	6.92
Number of years in public health practice (any agency, any position)	9.39	8.27
**Characteristic**	**Median**	**Min**	**Max**
Year born (*n* = 71)	1978	1958	2001

### Post-session surveys

On average, 55 participants completed the post-session survey among the six ECHO sessions (range 46–58). Participants were asked to respond to a series of statements using a 5-point Likert scale with 1 being strongly disagree and 5 being strongly agree. On average, 88% of participants agreed or strongly agreed that the ECHO sessions and speakers met their expectations, they learned something new, and the case-based discussions were impactful to their learning (Table 3). Participants were also asked to respond to a series of statements using a 4-point Likert scale with 1 being not at all and 4 being very likely. Approximately 98% of respondents reported being somewhat or very likely to use or share the information they learned from the ECHO series (Table 4).

**Table 3:** ECHO participant satisfaction with ECHO sessions by session.

**Session number**	**Overall, this ECHO session met my expectations** ^*^	**The speaker for this session met my expectations**	**This session's case-based discussion was impactful on my learning**	**I learned something new in this session**
1 (*n* = 59)	4.19	4.31	4.10	4.42
2 (*n* = 58)	4.33	4.40	4.30	4.33
3 (*n* = 58)	4.31	4.24	4.28	4.23
4 (*n* = 46)	4.02	4.09	4.02	4.07
5 (*n* = 54)	4.11	4.13	4.15	4.07
6 (*n* = 53)	4.21	4.21	4.28	4.23
All sessions average	**4.20**	**4.24**	**4.19**	**4.23**

^*^Scores range from 1 to 5. 1-Strongly disagree, 2-disagree, 3-neither agree nor disagree, 4-Agree, 5-Strongly agree.

**Table 4:** ECHO participant likelihood of sharing or using information learned from each session in the series.

**Session number**	**How likely are you to use the didactic information you learned in this session?**	**How likely are you to share the information you have learned so far in the ECHO series with your colleagues?**
1 (*n* = 59)	3.56	3.63
2 (*n* = 58)	3.64	3.69
3 (*n* = 57)	3.40	3.58
4 (*n* = 46)	3.41	3.50
5 (*n* = 54)	3.44	3.46
6 (*n* = 53)	3.47	3.51
All sessions average (*n* = 327)	**3.49**	**3.57**

^*^Scores range from 0 to 4. 1-Not at all, 2-not very likely, 3- somewhat likely, 4-very likely.

### Post-series survey

Overall, 94% of the 65 participants who completed the post-series survey (*n* = 61) attended at least four of the six ECHO sessions. Before the series, over half of the respondents collaborated with other public health departments at least monthly (*n* = 40/74; 54%). After the series, approximately 83% of participants (*n* = 54/65) increased how often they discussed dementia risk reduction with other health department colleagues outside of their specific jurisdiction, and 94% of participants (*n* = 61/65) increased how often they collaborated with other public health departments. Respondents who did not report collaborating noted time constraints/schedules, distance, lack of contact information for other health departments, and differences in operations as barriers to collaboration.

When asked how soon they would implement something they learned from the ECHO series, 40% of respondents (*n* = 26/65) said they would implement something they learned immediately and 18% (*n* = 12/65) said they would implement something within the next 30 days. Specific areas of implementation included educating staff, patients, and community members on dementia risk reduction, brain health, and modifiable risk factors; and disseminating health education materials to different age groups (e.g., older adults individuals, young adults, and the youth population). Additionally, participants noted that they would provide resource lists to providers and patients, work with community partners to provide education at centers for older adults centers, and incorporate dementia risk reduction education into existing programs.

**Figure 1:**
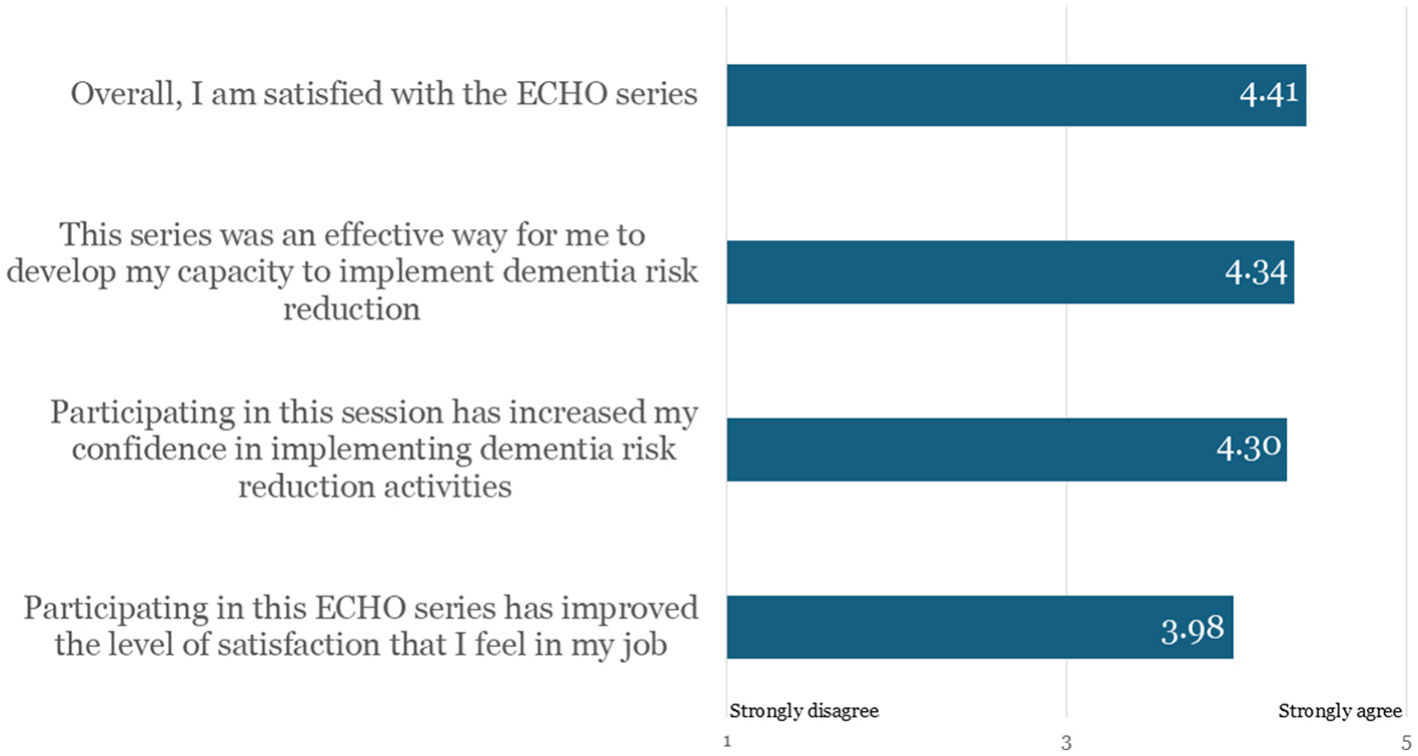
Participant satisfaction with the ECHO series overall (*n* = 65).

Participants were asked to respond to a series of statements on participant satisfaction using a 5-point Likert scale. On average, participants were highly satisfied with the series with average scores ranging from 3.98 to 4.41 (Figure 1). However, through open-ended responses, some participants suggested opportunities for program improvement, including more engaged presentations and education on early intervention, break-out sessions for discussions, expansion of the social determinants of health/equity session, and inclusion of the whole health department. Additionally, respondents would like examples of how to discuss Alzheimer's disease with patients exhibiting early symptoms of the disease.

### Post-series paired sample *t*-test

To understand changes in public health professionals' perspective, knowledge, and confidence, analysis was conducted among survey responses from participants who completed both the pre-series and post-series surveys (*n* = 60 matched pairs). On average, across respondents to both the pre- and post-series surveys, there was an 11% increase in reported agreement with dementia as a public health issue and a 10% increase in reported agreement that dementia risk reduction plays a role in public health.

For the paired samples t-test analysis, Participants were asked to respond to a series of statements using a 5-point Likert scale. The difference between the post-series and pre-series Likert rating was averaged across all participants to calculate the mean difference score. As shown in Table 5, all paired samples t-test analysis showed statistically significant positive change over time related to public health professional perspective, knowledge gain, and increased confidence. Some of the largest changes were related to existing resources or tools to assist with implementation and action plans (mean difference = 1.29), lifelong dementia risk reduction methods (mean difference = 1.27), data use by public health agencies to address modifiable risk factors (mean difference = 1.24), and how engaging community partners can help public health agencies address modifiable risk factors (mean difference = 1.24).

**Table 5:** Change in public health professional perspective, knowledge, and confidence between pre and post-series surveys.

**Variable**	***n***	**Mean difference**	**Standard error**	***t*-statistic**	***p* value**
**Public health practitioner perspective**					
Dementia risk reduction is a public health issue.	60	0.48	0.15	3.28	0.002
Dementia risk reduction plays a role in public health.	60	0.43	0.17	2.52	0.01
**Knowledge change**					
Dementia as a public health issue.	60	0.95	0.13	7.37	<0.001
Role of public health agencies in dementia risk reduction.	60	1.02	0.14	7.28	<0.001
Tools for public health agencies to lessen the overall burden of dementia.	60	1.05	0.15	7.03	<0.001
Lifelong dementia risk reduction methods.	60	1.27	0.15	8.55	<0.001
Modifiable risk for dementia.	58	1.09	0.14	7.65	<0.001
Social determinants of health related to brain health and dementia.	58	1.09	0.12	8.78	<0.001
Data use by public health agencies to address modifiable risk factors.	59	1.24	0.17	7.21	<0.001
Engaging community partners can help public health agencies address modifiable risk factors.	59	1.24	0.15	7.96	<0.001
Existing resources or tools to assist with implementation and action plans.	59	1.29	0.16	7.85	<0.001
Developing and implementing action plans to address reduction strategies.	58	1.17	0.16	7.26	<0.001
**Confidence levels**					
Getting leadership buy-in/support from my organization to address dementia risk reduction.	59	0.76	0.19	3.90	<0.001
Forming new partnerships with other organizations, community leaders, and/or people with lived experience to address dementia risk reduction.	59	0.69	0.17	4.01	<0.001
Strengthening existing partnerships with other organizations, community leaders, and/or people with lived experience to address dementia risk reduction.	59	0.69	0.15	4.47	<0.001
Building dementia risk reduction capacity within my organization.	59	0.75	0.17	4.29	<0.001
Building dementia risk reduction capacity across partner organizations.	59	0.69	0.17	4.10	<0.001

### Hub member focus group

ECHO series Hub members unanimously reported the program was both highly successful and valuable. They noted that having participant and contextual information in advance, a curricula relevant for multigenerational households, and age-inclusive ADRD public health education made their sessions effective learning environments.

Hub members also encountered a few challenges related to course materials, enrollment, and post-series follow-up. They shared that there was so much content, it was challenging to fit everything into one session, and occasionally the case study examples did not align with the didactic sessions. Hub members also wanted opportunities to follow up with the participants on strategies and actions learned in the ECHO series. Suggested improvements for future ECHO series included better alignment with didactic sessions and arranging a meeting between the Department of Health and the Hub presenters to ensure every participant is prepared for the session/series.

### Participant focus group

Participants reported that the ECHO series was impactful toward their understanding and awareness of the risk factors for dementia. They found the educational materials essential to the success of the program. The ability to review slides and recordings after sessions allowed for thorough engagement with the material and encouraged resource-sharing. The primary challenge participants reported was time conflicts for meetings; however, session recordings and accessible materials provided an apt solution.

Following the ECHO series, participants noted an increase in knowledge and awareness about dementia and dementia risk reduction. Several participants mentioned the ECHO series changed their misperception that dementia is a normal part of aging and increased their awareness of the dementia risk factors that overlap with other chronic disease conditions. This understanding allowed them to incorporate dementia risk reduction into existing prevention work at their organizations.

While most participants had not yet formally implemented dementia risk reduction into their work, participants mentioned their intention to do so. Participants planned to disseminate materials to nurse officials, patients, community members, staff within their health departments, and caregivers. They also mentioned plans to attend future trainings, learning collaboratives, and other events to develop partnerships and understand how best to educate their community. Furthermore, participants identified health educators as a suitable source to disseminate information, as they serve as primary representatives of health departments, and one participant had already started disseminating materials to their team of health educators. Participants suggested developing an ECHO Series tailored to health educators. Participants noted the strong leadership support in Tennessee from commissioners and others in leadership roles, which left them feeling supported to start incorporating these ideas into their work.

Participants offered suggestions to improve the ECHO program in the future, such as developing tailored resources and materials for different audiences (clinical staff, community members, administrative staff, etc.) to maximize effectiveness. They also suggested shorter didactic sessions to create more space for interaction among participants through roundtables or breakout rooms. Further emphasizing the desire for peer engagement, participants advised creating a post-program learning community so they could share ideas, problem solve and maintain connections. Participants additionally proposed inviting alumni from the pilot cohort to present during the next series, as they saw value in sharing examples of successful implementation with each other.

## Discussion

### Impact and replicability

Since the completion of this pilot, The Alzheimer's and Dementia Care ECHO Program for Public Health Professionals has been replicated in two additional states. As the ECHO model continues to be refined and replicated in other states, it has the potential to significantly improve public health outcomes and increase action around the modifiable risk factors for dementia. Leveraging the Alzheimer's Association ECHO Superhub, this curriculum is now available for adoption by other ECHO hubs and will continue to be refined and implemented by the Center and the Association ECHO team. The results presented in this manuscript demonstrate how the ECHO model can be adapted to train public health professionals in a cost-effective and impactful way. By leveraging technology, the ECHO model offers a cost-effective way for public health professionals to increase knowledge, confidence, and collaboration between and among state and local health departments. This approach could be used for multiple topics to address complex public health challenges. Specifically for dementia risk reduction, the ECHO training model offered an opportunity to increase conversation, knowledge, and confidence across the state of Tennessee. Using the ECHO “all teach, all learn” model allowed for discussion of implementation ideas and collaborative problem solving among state and local health department teams.

The Tennessee Department of Health played a pivotal role in this process, encouraging participants to pursue Age-Friendly Public Health System (AFPHS) recognition. This framework ensures that public health systems meet the needs of older adults, promoting healthier aging and improving the quality of life for older adults. Strong leadership from the Tennessee Department of Health also resulted in increased local action, with multiple health departments participating in collaborative programs. The public health approach to dementia risk reduction is not widely known in the public health community. The ECHO model enables broad reach by facilitating rapid dissemination of knowledge and resources about dementia risk reduction to diverse communities. To take a public health approach to dementia, public health professionals must first be aware of the strategies and be confident in their ability to implement these strategies in their communities. Programs like the Alzheimer's and Dementia Care ECHO Program for Public Health Professionals offer a forum to increase public health professional's knowledge and confidence and allow discussion and opportunities for immediate action, as was seen by the 60% of attendees reporting learning something they would implement immediately or in the next 30 days. Further improvements can be made to the program by addressing challenges, such as extending their duration while reducing frequency to allow for deeper exploration of content and more meaningful discussion. Incorporating Zoom breakout rooms can also enhance participant engagement by fostering small-group interaction and peer-to-peer learning. Additionally, addressing challenges like misalignment between the case example and didactic session, and strengthening peer engagement through post-program learning communities, will further support the program's effectiveness and impact. Through such improvements, the ECHO model can further improve its efficacy and scalability.

By collaborating with leading scientific experts and developing meaningful partnerships with health departments nationwide, the ECHO model will continue to be a bridge from research to practice, serving as a critical resource to increase the knowledge, confidence and capacity of the public health workforce to address dementia risk. As public health efforts on dementia risk reduction grow and expand, promoting brain health and encouraging the adoption of healthy habits across the lifespan must become a priority. State and local health departments are a critical partner to share this information and integrate dementia risk reduction into state and local health priorities.

## Data Availability

The raw data supporting the conclusions of this article will be made available by the authors, without undue reservation.
